# Combinations of PRI-724 Wnt/β-Catenin Pathway Inhibitor with Vismodegib, Erlotinib, or HS-173 Synergistically Inhibit Head and Neck Squamous Cancer Cells

**DOI:** 10.3390/ijms241310448

**Published:** 2023-06-21

**Authors:** Robert Kleszcz, Mikołaj Frąckowiak, Dawid Dorna, Jarosław Paluszczak

**Affiliations:** Department of Pharmaceutical Biochemistry, Poznan University of Medical Sciences, 4, Święcickiego Str., 60-781 Poznań, Poland; mikfrackowiak97@gmail.com (M.F.); dawid.dorna97@gmail.com (D.D.); paluszcz@ump.edu.pl (J.P.)

**Keywords:** Wnt pathway, hedgehog pathway, β-catenin, PRI-724, vismodegib, erlotinib, HS-173, head and neck cancer, cancer stem cells, synergism

## Abstract

The Wnt/β-catenin, EGFR, and PI3K pathways frequently undergo upregulation in head and neck squamous carcinoma (HNSCC) cells. Moreover, the Wnt/β-catenin pathway together with Hedgehog (Hh) signaling regulate the activity of cancer stem cells (CSCs). The aim of this study was to investigate the effects of the combinatorial use of the Wnt/β-catenin and Hh pathway inhibitors on viability, cell cycle progression, apoptosis induction, cell migration, and expression of CSC markers in tongue (CAL 27) and hypopharynx (FaDu) cancer cells. Co-inhibition of Wnt signaling with EGFR or PI3K pathways was additionally tested. The cells were treated with selective inhibitors of signaling pathways: Wnt/β-catenin (PRI-724), Hh (vismodegib), EGFR (erlotinib), and PI3K (HS-173). Cell viability was evaluated by the resazurin assay. Cell cycle progression and apoptosis induction were tested by flow cytometric analysis after staining with propidium iodide and Annexin V, respectively. Cell migration was detected by the scratch assay and CSC marker expression by the R-T PCR method. Mixtures of PRI-724 and vismodegib affected cell cycle distribution, greatly reduced cell migration, and downregulated the transcript level of CSC markers, especially *POU5F1* encoding OCT4. Combinations of PRI-724 with erlotinib or HS-173 were more potent in inducing apoptosis.

## 1. Introduction

Head and neck squamous cell carcinomas (HNSCCs) are a diverse group of tumors that share common clinical risk factors, including smoking tobacco, often with concomitant abuse of high-proof alcohol, or oncogenic virus infection (mainly the human papilloma virus, HPV, or the Epstein–Barr virus, EBV). On the other hand, HNSCCs are characterized by heterogeneous genetic and molecular abnormalities, including the most frequently occurring mutations of *TP53*, and mutations/alterations in other cell cycle regulators or epigenetic changes [1]. According to the Global Cancer Statistics 2020, new cases of lip and oral cavity cancers amounted to 2.0%, followed by other head and neck localizations: larynx (1.0%), nasopharynx (0.7%), oropharynx (0.5%), and hypopharynx (0.4%). Lip and oral cavity cancers had the highest incidence rate per 100,000 in the male population of Melanesia (22.2), South Central Asia (13.3), and Eastern Europe (9.2) [2].

The rapid development of pharmaceutical and medical sciences has resulted in translational ‘bench to bedside’ studies allowing for more robust treatment of oncological disorders. Since epidermal growth factor receptor (EGFR) is commonly overexpressed among HNSCC patients, EGF signaling was thought to be a good target for HNSCC treatment. Unfortunately, despite the dysregulation of EGFR expression reaching 90% of cases, only 10–30% of patients respond to monotherapy based on EGFR inhibitors like cetuximab, a chimeric mouse-human IgG1 antibody against the extracellular domain of EGFR [3]. It has been suggested that EGFR-directed therapies can be improved by the concurrent targeting of EGFR-related PI3K/Akt signaling and by searching for combination therapies with other molecular targets important for the growth of HNSCC [4,5]. In this regard, our earlier study showed that the effects of erlotinib, a small-molecule inhibitor of EGFR, can be augmented by the concurrent use of inhibitors of KDM4 or KDM6 histone lysine demethylases [6].

In previous years, an idea arose to pharmacologically target/inhibit the Wnt canonical (β-catenin-dependent) signaling pathway because its dysregulation, also based on epigenetic mechanisms, can be observed as early as at the pre-cancerous stage, like oral submucous fibrosis [7]. The dysregulation in Wnt/β-catenin signaling stimulates HNSCC cell proliferation, supports the avoidance of apoptosis, and improves cell migration [8,9]. Wnt canonical signaling can be inhibited at various levels of signal transduction, starting from the maturation of Wnt ligands, through pathway activation at the cell membrane, further secondary transduction of signals through the cytoplasm, and finally to the modulation of TCF/LEF transcription factor activity in the nucleus. In fact, both synthetic and natural compounds can affect particular targets [10,11,12,13,14]. Our previous research on six HNSCC cell lines allowed for the identification of two suitable protein targets: Porcupine, the *O*-acyltransferase required for the maturation of Wnt ligands, and the interaction of β-catenin with CBP, which is crucial for the activation of transcription of Wnt signaling target genes [15]. In the first case, the inhibition has an influence on both canonical and non-canonical Wnt signaling, but the second target selectively attenuates the Wnt/β-catenin pathway.

Stem cells (SCs) play an indispensable role in hierarchical development during embryogenesis and remain active in adult tissues to maintain their self-renewal ability. Moreover, it is now widely acknowledged that tumors contain a population of so-called cancer stem cells (CSCs), which can support phenotypically diverse tumor mass growth and create resistance to standard therapy [16]. The presence of CSCs was also detected in HNSCCs, and targeting this subpopulation of cells has become a potential new therapeutic strategy [9,10,14,17,18]. Importantly, Wnt/β-catenin pathway inhibition had anti-stemness potential in HNSCC cells [19]. Indeed, this pathway maintains the activity of both normal and cancer stem cells [10]. Hedgehog (Hh) is another pathway responsible for the viability and function of normal SCs and CSCs, and it is active in different types of head and neck cancers [20]. Thus, Wnt/β-catenin and Hh pathways can both be involved in the promotion of CSCs in HNSCC, which can potentiate the development of cancer. There is crosstalk between these pathways, and recently, the GSK-3β kinase activity state was shown to be important for both Wnt and Hh signaling control [21]. Moreover, EGFR and PI3K/Akt signaling are able to accelerate the tumorigenic potential of HNSCC cells and present crosstalk with Wnt and Hh pathways [22]. Currently, there are limited data about the potential usefulness and benefits of targeting the Hh pathway in HNSCC. Vismodegib (GDC-0449) is a small, orally administrable molecule that inhibits the Hh pathway and was approved by the U.S. Food and Drug Administration for the treatment of basal cell carcinoma (BCC) [23]. However, evidence regarding its biological activity in HNSCC is scarce.

Considering the above data and our previous research, we aimed to analyze the influence of Wnt/β-catenin pathway inhibition by PRI-724 and Hedgehog pathway inhibition by vismodegib, individually and in combinations, on the viability, cell cycle progression, apoptosis induction, and cell migration of squamous cell carcinoma of the tongue (CAL 27) and hypopharynx (FaDu) cancer cells. The results were compared to the effects of the co-inhibition of Wnt/β-catenin and better-known molecular targets in HNSCC, particularly the EGF receptor (targeted by erlotinib) and PI3 kinase (targeted by HS-173). Furthermore, we evaluated the influence of PRI-724 and vismodegib on the expression of selected genes related to CSC to preliminarily check the potential of Wnt and Hh pathway inhibition in modulating their levels in CAL 27 and FaDu cells. All combinations demonstrated beneficial effects in HNSCC cells, but the profile of biological activity differed. The mixtures of PRI-724 and vismodegib were especially interesting because of their ability to accumulate cells in the G1/G0 phase, greatly reduce cell migration, and downregulate the transcript level of genes expressed by cancer stem cells.

## 2. Results

### 2.1. Expression of the Components of the Wnt/β-Catenin Pathway Is Increased in Higher Grade HNSCC Tumors

**Figure 1:**
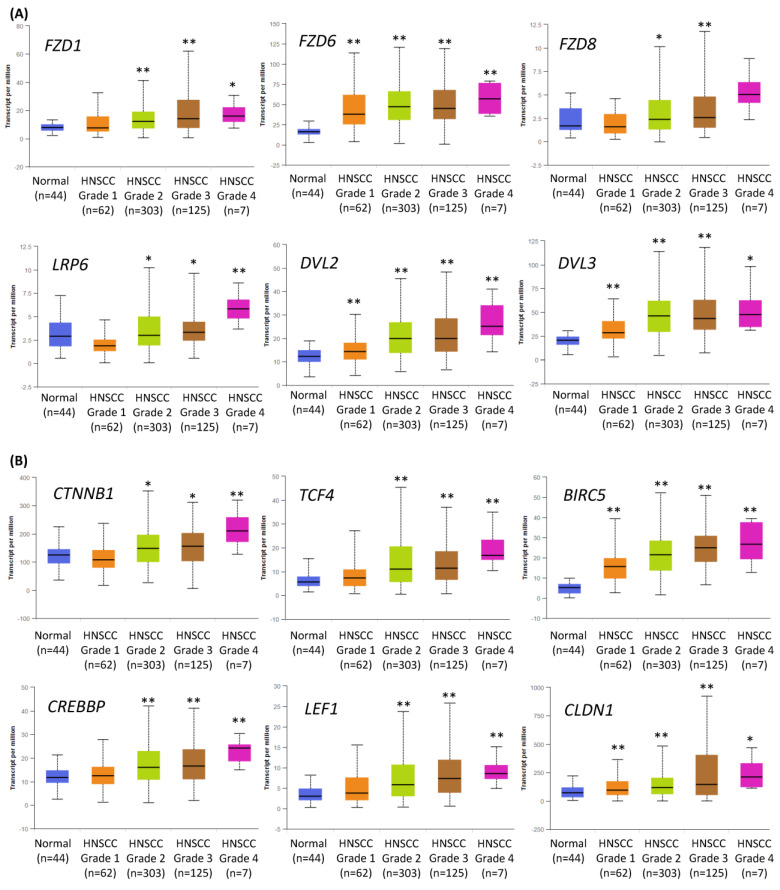
The results of the analysis of Wnt/β-catenin pathway-related gene expression data available from The Cancer Genome Atlas using the UALCAN tool. The differences in the level of gene expression depending on tumor grade (grade 1—well-differentiated, grade 4—undifferentiated), compared with normal epithelium, are shown. The asterisk (*) above the bar denotes statistically significant changes in comparison to normal control samples, * *p* < 0.05, ** *p* < 0.01. (**A**) Exemplary genes related to the transduction of the signal from Wnt ligand to cytoplasm: Frizzled receptors (*FZD1*, *FZD6*, *FZD8*), *LRP6* co-receptor, and cytoplasmic protein Dishevelled (*DVL2*, *DVL3*) that relays Wnt signals from receptors to downstream effectors. (**B**) Genes related to the nucleic part of Wnt signal transduction: β-catenin (*CTNNB1*) creating complexes with CREB binding protein (*CREBBP*), exemplary transcription factors (transcription factor 4—*TCF4*, lymphoid enhancer binding factor 1—*LEF1*), and target genes of Wnt/β-catenin pathway (survivin—*BIRC5*, claudin 1—*CLDN1*).

We have previously tested the value of the inhibition of canonical Wnt signaling to treat HNSCC cells [15], and we hypothesized that the effects of Wnt pathway inhibition could be further augmented by co-treatment with chemicals targeting other key signaling pathways. Indeed, the analysis of TCGA data corroborated that the Wnt pathway is frequently dysregulated in HNSCC, and the changes increase with tumor grade (Figure 1). Figure 1A contains data on the expression of several genes related to the first step of Wnt/β-catenin signaling activation. These initial events are based on the attachment of the Wnt ligand to the Frizzled (FZD) receptor. Further, a co-receptor (LRP) and the cytoplasmic protein Dishevelled (DVL) bind together to form a Wnt-FZD-LRP-DVL complex, which leads to downstream effects.

Figure 1B presents data on the expression of the essential elements of the nuclear part of canonical Wnt signal transduction, especially β-catenin and CREB binding protein (CBP). PRI-724, the small-molecule inhibitor of the Wnt/β-catenin pathway used in this study, blocks the interaction between β-catenin and CBP, which is crucial for their transcriptional activity. A complex of β-catenin and CBP binds to and activates transcription factors (TCF1, TCF3, TCF4, LEF1) and promotes the expression of canonical Wnt signaling target genes, e.g., the anti-apoptotic survivin (*BIRC5*) and claudin 1 (*CLDN1*)—a component of tight junction complexes.

In general, the expression of Wnt/β-catenin pathway-related genes was higher in HNSCC samples than in normal epithelium samples and showed growth with the increase in tumor grade. This suggests that the progression of HNSCC relies on the elevated activity of Wnt/β-catenin signaling. Thus, in this study, the inhibition of the Wnt/β-catenin pathway was considered the central and starting point of co-treatment strategies of HNSCC cells with combinations of PRI-724 and inhibitors targeting other molecular pathways.

### 2.2. Vismodegib and PRI-724 Reduce the Viability of CAL 27 and FaDu Cells

**Figure 2:**
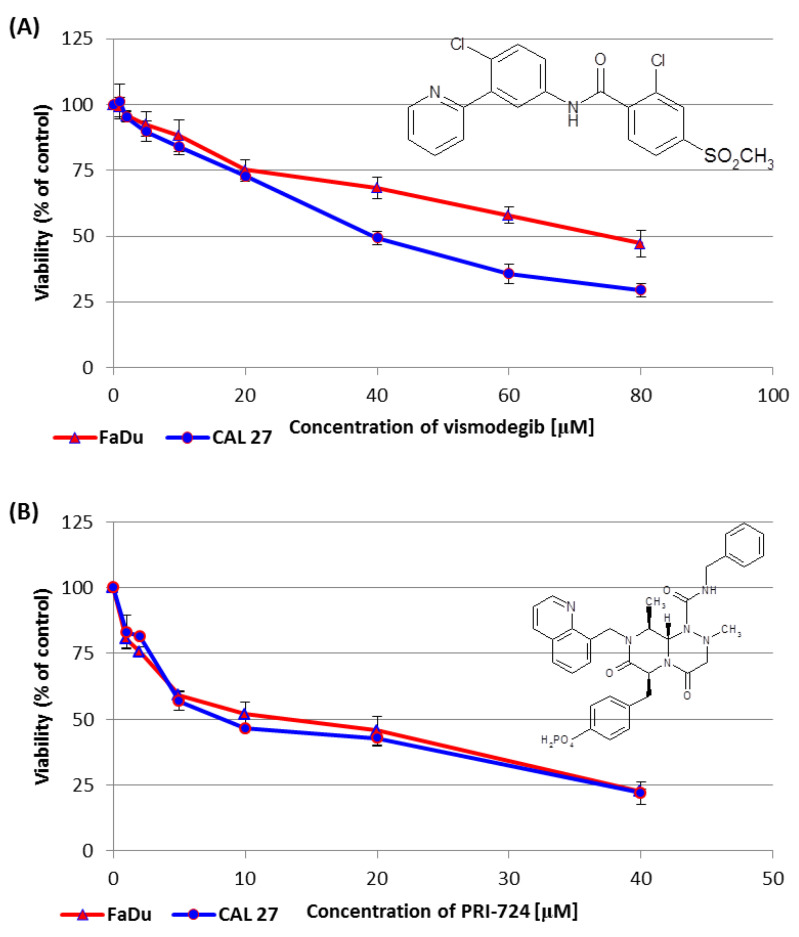
The effect of vismodegib (**A**) and PRI-724 (**B**) on the viability of CAL 27 and FaDu cells after 48 h of incubation, as determined by the resazurin assay. The chemical structure of the compounds is also presented. The results are the mean values from three independent experiments with four technical repeats ± SD.

We performed the resazurin assay to establish the influence of vismodegib, an inhibitor of the Hedgehog pathway, on the viability of CAL 27 cells (tongue cancer) and FaDu cells (hypopharyngeal cancer). The sub-toxic effect (IC25) was reached for both CAL 27 and FaDu cells at a similar level. However, at concentrations above 20 µM CAL 27 cells were more sensitive to vismodegib in comparison to FaDu cells (Figure 2A).

In the same assay, PRI-724 showed similar effects on both tongue and hypopharynx cancer cells. However, around the IC50 value, once again, CAL 27 cells were more affected than FaDu cells (Figure 2B).

Table 1 shows the concentrations of vismodegib and PRI-724, which reduced cell viability by 25% (IC25) or by 50% (IC50) in CAL 27 and FaDu cells. The IC25 and IC50 values for erlotinib and HS-173 were established in our previous research [6] and are also shown in Table 1 with an asterisk.

### 2.3. Combinations of PRI-724 with Other Inhibitors Present Better Synergistic Effects in FaDu Cells

In this research, we wanted to compare the effects of the inhibition of Wnt canonical signaling by PRI-724 with the inhibitors of three other important pathways—Hedgehog, EGFR, and PI3K. Firstly, we evaluated the effects of the combinations of inhibitors on the viability of CAL 27 and FaDu cells. Detailed information about the concentrations and proportions of chemicals as well as the effects on viability reduction can be found in Supplementary Table S1.

The collected data were analyzed using the CompuSyn software, version 1.0 (downloaded from the website www.combosyn.com on 31 May 2021). This software can be used to calculate single-drug and drug-combined pharmacodynamics. The unique mathematical system analysis for dose-effect dynamics led to the discovery of the median-effect equation (MEE), the unified biodynamics, pharmacodynamics, and bioinformatics general principle. This unified dynamics algorithm allows using a few dose data points to create an automated quantitative simulation, unlike the traditional, expensive experimental approach that uses many dose data points to fit an empirical curve.

The results of analyses refer to the ‘Combination Index’ (CI). There are three general conclusions concerning the CI value. CI = 1 denotes that the experimental result of the drug combination compared to the effects of individually used compounds is additional (additive effect). A CI value below 1 reflects better experimental effects than just the mathematically calculated addition of single compound activity and is called synergism. Conversely, a CI above 1 suggests the loss of some of the effects associated with the action of individual compounds (antagonism).

When the proportions between concentrations of mixed compounds are changeable, only the CI for a particular mixture can be calculated. However, when this proportion is stable, e.g., 1:1, CompuSyn software creates plots showing CI (the character of interaction) in a broad concentration range, extrapolating experimental data. On the 0X axis, the parameter ‘fraction affected’ (Fa) is used. Fa means the intensity of the effect, in this case, the reduction of cell viability. Fa = 0 denotes a lack of viability reduction, and Fa = 1 is a synonym for total cell death.

**Figure 3:**
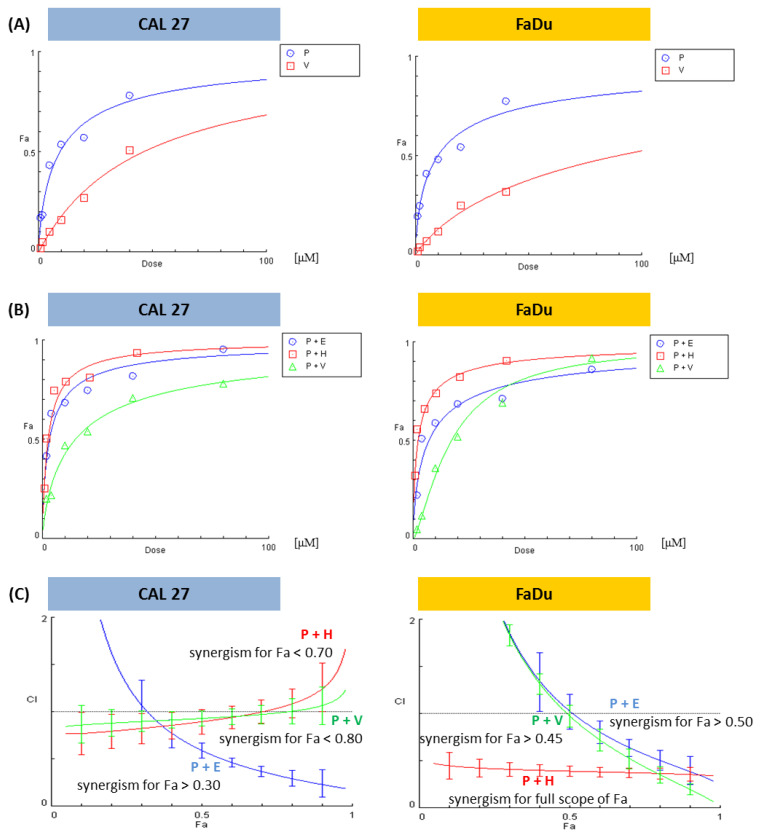
The results of the analysis of the combinatorial effects between PRI-724 and vismodegib, erlotinib, or HS-173 on cell viability by the resazurin assay. CAL 27 and FaDu cells were treated with increasing concentrations (Supplementary Table S1) of the individual compounds and their combinations for 48 h. Control cells were treated with vehicle (DMSO). Mean values from three independent experiments were used in calculations using the CompuSyn software (version 1.0). Dose-effect curves for individual compounds (**A**) or their combinations (**B**) were generated. Plots (**C**) represent the evaluation of the Combination Index (CI), where CI < 1 (the area below the dotted line in plots) denotes a synergistic effect between compounds. Fa—Fraction affected: viability reduction from lack of effect (0) to maximal effect (1; viability = 0%), E—erlotinib, H—HS-173, P—PRI-724, V—vismodegib.

We showed dose–effect curves for individual PRI-724 and vismodegib, which were evaluated by the CompuSyn software for the first time (Figure 3A). Figure 3B presents dose-effect curves for PRI-724 combined with either vismodegib, erlotinib, or HS-173. The Combination Index calculated for each mixture in the form of plots showing the character of interaction between two inhibitors in the function of Fa is shown in Figure 3C.

Based on the analysis of the interaction between the compounds, it can be stated that the combination of PRI-724 and HS-173 synergistically reduced the viability of FaDu cells (Figure 3C). In other cases, synergism was detected for a part of the effect range. Two mixtures in FaDu cells needed higher concentrations of compounds to reach synergistic effects, namely, Fa > 0.45 for the combination with vismodegib and Fa > 0.50 for the combination with erlotinib. In CAL 27 cells, the best results were observed for the mixture of PRI-724 and erlotinib, where synergism appeared for Fa > 0.30. In turn, data for the combinations of PRI-724 with vismodegib (Fa < 0.80) and HS-173 (Fa < 0.70) suggest weak synergism below the indicated Fa.

In all further experiments, we used the concentrations of compounds presented in Table 1.

### 2.4. Signaling Inhibitors Affect Proliferation Mostly by Cell Cycle Arrest in the G1/G0 Phase

**Figure 4:**
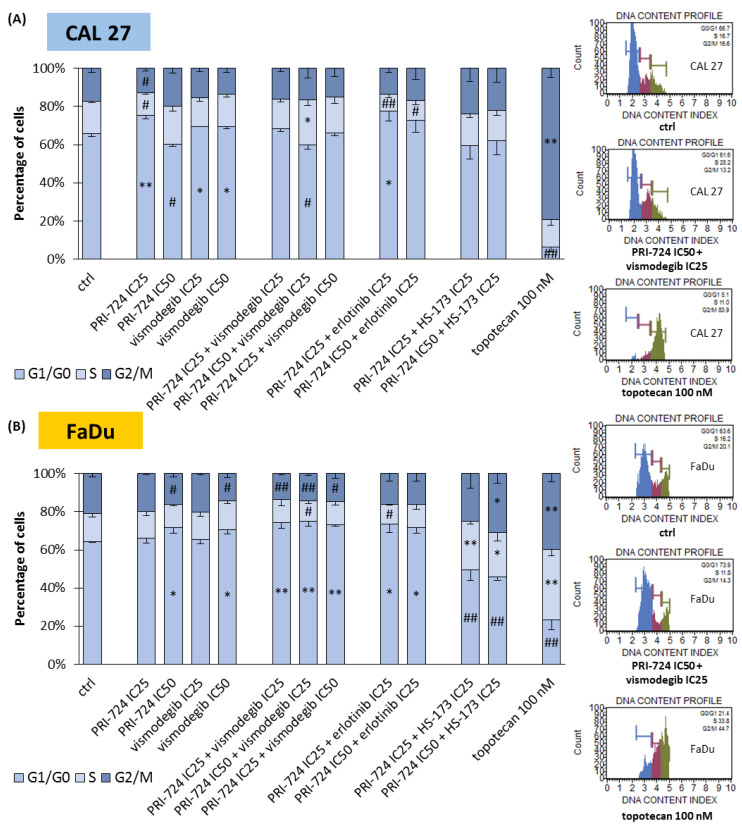
The effect of PRI-724, vismodegib, erlotinib, HS-173, and their combinations on the cell cycle distribution in CAL 27 (**A**) and FaDu (**B**) cells after 48 h of incubation. The cell cycle distribution (G1/G0, S, and G2/M phases) was measured by flow cytometric analysis of cells after propidium iodide staining. DMSO-vehicle-treated cells were used as a negative control, while topotecan (100 nM) was used as a positive control for cell cycle arrest. Exemplary flow cytometry plots are shown on the right side. Mean values ± SD from three independent experiments are shown. The asterisk (*) inside the bars denotes a statistically significant increase in cell population between the analyzed compound used alone or in combination, and the control (ctrl), * *p* < 0.05, ** *p* < 0.01; (#) inside the bars denotes a statistically significant decrease in cell population between the analyzed compound used alone or in combination, and the control (ctrl), # *p* < 0.05, ## *p* < 0.01.

We performed an analysis of the cell cycle distribution of CAL 27 and FaDu cells to establish the possible influence of mixtures of chemicals on the induction of cell cycle arrest (Figure 4). In CAL 27 cells (Figure 4A), PRI-724 at IC25 and both concentrations of vismodegib induced the accumulation of cells in the G1/G0 phase. The same effect was observed previously for erlotinib [6]. Interestingly, the treatment of cells with PRI-724 at IC50 caused the opposite effect, i.e., the reduction of the G1/G0 population of CAL 27 cells. Such a result was also present in PRI-724 at IC50 combined with vismodegib at IC25, suggesting that the activity of Wnt/β-catenin inhibitor was dominant. However, both mixtures of erlotinib and PRI-724 were able to arrest cells in G1/G0 phases.

The accumulation of FaDu cells in G1/G0 phases was the main effect for all the combinations of PRI-724 with vismodegib and erlotinib (Figure 4B). A significant change for individually used PRI-724 or vismodegib was found only at IC50 concentrations. Surprisingly, mixtures of PRI-724 with HS-173 significantly reduced the G1/G0 population of cells with the concomitant accumulation of cells in S and/or G2/M phases, although HS-173 at IC25 [6] and PRI-724 at IC25 individually had no effect or even showed the opposite effect, like in the case of PRI-724 at IC50.

### 2.5. Combinations of PRI-724 with Erlotinib or HS-173 Exert Better Pro-Apoptotic Effects

**Figure 5:**
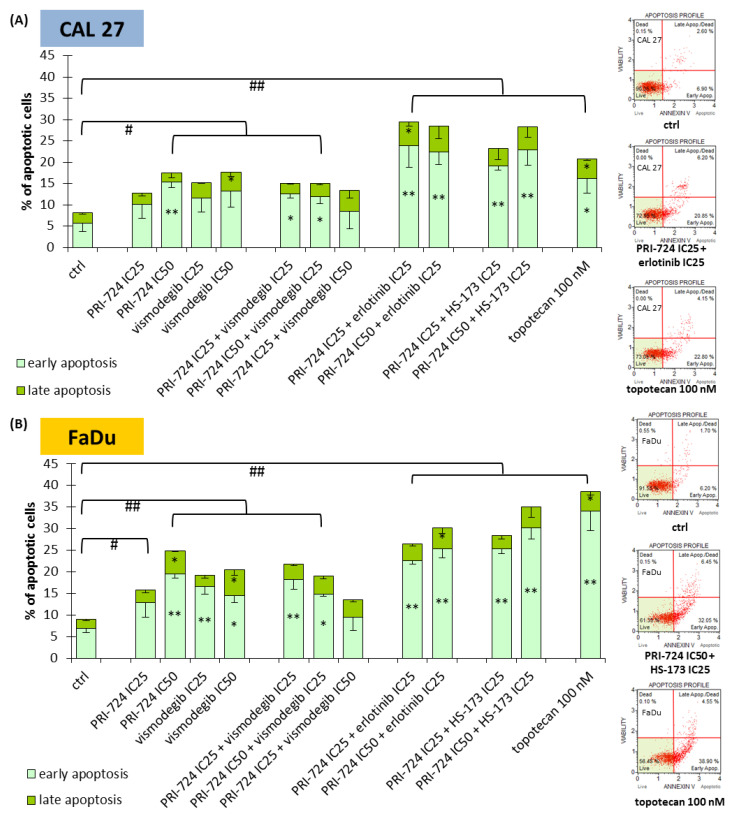
The effect of PRI-724, vismodegib, erlotinib, HS-173, and their combinations on the induction of apoptosis in CAL 27 (**A**) and FaDu (**B**) cells after 48 h of incubation. The apoptotic cells were detected by flow cytometric analysis after Annexin V and 7-Aminoactinomycin D staining. DMSO-vehicle-treated cells were used as a negative control, while topotecan (100 nM) was used as a positive control for apoptosis induction. Exemplary flow cytometry plots are shown on the right side. Mean values ± SD from three independent experiments are shown. The asterisk (*) inside the bars denotes a statistically significant increase in cell population between the analyzed compound used alone or in combination, and the control (ctrl) for early or late apoptotic cells, * *p* < 0.05, ** *p* < 0.01; (#) above the bars denotes a statistically significant increase in total apoptotic cells between the analyzed compound used alone or in combination, and the control (ctrl), # *p* < 0.05, ## *p* < 0.01.

In the next step, we evaluated whether the changes in cell cycle distribution were also followed by the induction of apoptosis (Figure 5). Both CAL 27 and FaDu cells were susceptible to a slight induction of apoptosis after individual exposure to PRI-724 and vismodegib, except PRI-724 at IC25 in CAL 27 cells. The same scale of action was previously observed for erlotinib and HS-173 [6]. The combination of PRI-724 with vismodegib failed to improve the effects. In turn, combinations of PRI-724 with erlotinib and HS-173 enriched the population of apoptotic cells. Discrimination between early and late phases of apoptosis points to the appearance of the early stages of apoptosis after 48 h of incubation with the evaluated compounds, including a 100 nM concentration of topotecan—the positive control for apoptosis.

### 2.6. PRI-724 Combined with Vismodegib or Erlotinib Synergistically Reduced Cell Migration

**Figure 6:**
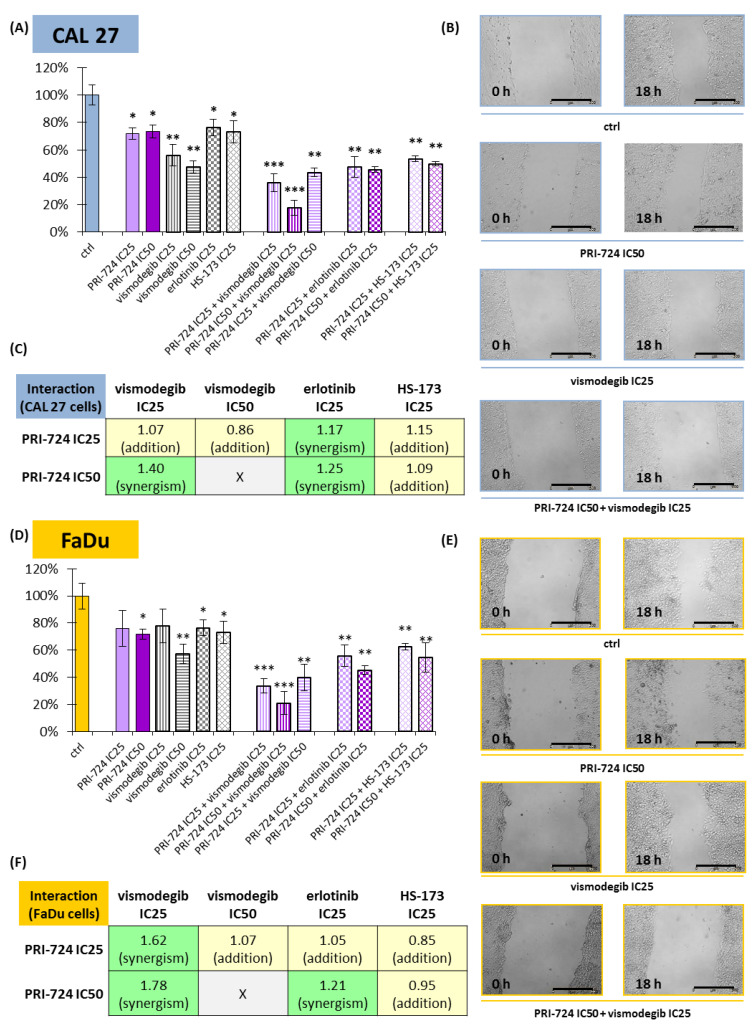
The scratch assay (cell migration) was performed for PRI-724, vismodegib, erlotinib, HS-173, and their combinations in CAL 27 (**A**–**C**) and FaDu (**D**–**F**) cells. Results (mean values ± SD) were calculated from four experiments (**A**,**D**). The asterisk (*) above the bars denotes a statistically significant decrease in migration rate between the analyzed compound used alone or in combination, and the control (ctrl), * *p* < 0.05, ** *p* < 0.01, *** *p* < 0.001. Exemplary microscopic images of the scratch, which were taken at the beginning and after 18 h of incubation with the compounds, are shown (**B**,**E**). The scale bar represents 500 μm. The assessment of the type of interaction between compounds using the modified Bürgi formula is shown (**C**,**F**). The addition of effects (0.85 ≤ *q* ≤ 1.15) is highlighted in yellow, while synergistic effects (*q* > 1.15) are highlighted in green.

Besides cell cycle arrest and the induction of apoptosis, the inhibition of signaling pathways can reduce the migration rate of cancer cells. We introduced the scratch assay to check the potential benefits of combining PRI-724 with other inhibitors (Figure 6).

All individually used compounds decreased cell migration in both CAL 27 and FaDu cells, except PRI-724 and vismodegib at IC25 concentrations in FaDu cells (Figure 6D). Notably, strong effects, with a >40% reduction in migration rate, were detected for vismodegib. Mixtures of chemicals significantly decreased the migration of CAL 27 and FaDu cells. Excellent results, with a reduction of approximately 80%, were obtained for the combination of PRI-724 at IC50 with vismodegib at IC25.

Next, we performed an analysis of the type of interaction between chemicals with regard to their effect on cell migration (Figure 6C,F). PRI-724 mixed with vismodegib or erlotinib had a synergistic effect on cell migration, most prominently in the case of the mixture with vismodegib at IC25 in FaDu cells (Figure 6F). The single use of vismodegib IC50 presented a beneficial effect, and its combination with PRI-724 IC25 led to only additive inhibition of migration. Similarly, the mixture of PRI-724 and HS-173 showed additive effects.

### 2.7. ALDH1A1, SOX2, and POU5F1 Genes Were Differentially Expressed among HNSCC Patients

**Figure 7:**
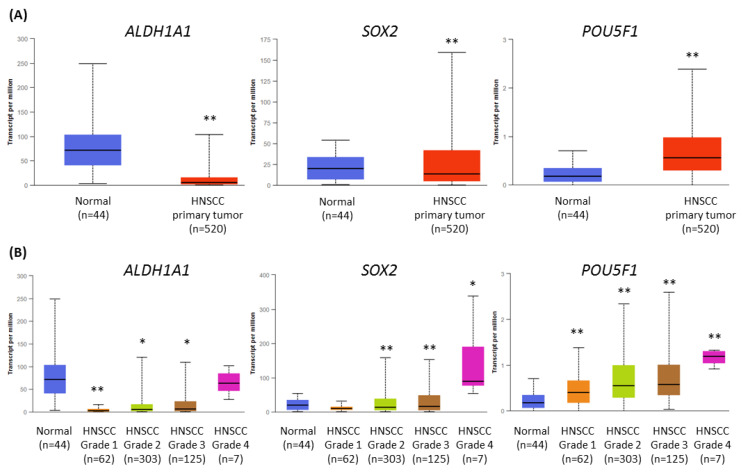
The results of the analysis of *ALDH1A1*, *SOX2*, and *POU5F1* gene expression data available from The Cancer Genome Atlas using the UALCAN tool. (**A**) The comparison of the level of expression between normal epithelium and HNSCC tumor samples. (**B**) The differences in the level of gene expression in relation to tumor grade (grade 1—well-differentiated, grade 4—undifferentiated), compared with normal epithelium. The asterisk (*) above the bar denotes statistically significant changes in comparison to normal control samples, * *p* < 0.05, ** *p* < 0.01.

Cancer stem cells (CSCs) play an important role in tumor development, survival, recurrence, and metastasis. Thus, we retrieved data from The Cancer Genome Atlas (TCGA) and analyzed the expression profile of ALDH1 (*ALDH1A1*), SOX2 (*SOX2*), and OCT4 (*POU5F1*) genes related to CSC in HNSCC, using the UALCAN tool [24,25]. Transcript levels were compared between HNSCC cases (*n* = 520) and normal epithelium samples (*n* = 44). The expression of *ALDH1A1* and *SOX2* was significantly lower in the population of HNSCC patients, but *POU5F1* showed upregulation (Figure 7A).

Next, we analyzed the differences in gene expression levels depending on tumor grade (Figure 7B). The transcript level of *ALDH1* was strongly reduced in grade 1 tumors, and in grade 4, the level was similar to normal tissue. The expression of *SOX2* was significantly lower in grades 2 and 3, and upregulated in grade 4. Finally, the transcript level of *OCT4* gradually increased from grade 1 to grade 4 tumors. In general, the expression of CSC-related genes was higher in histologically more advanced HNSCC cases, where the role of CSC is probably increasing.

### 2.8. Vismodegib Effectively Reduced the Expression of Cancer Stem Cells-Related Genes in CAL27 and FaDu Cells

Wnt and Hedgehog signaling pathways have an influence on the CSC population and can control each other’s activities. Thus, we decided to compare the activity of Wnt and Hedgehog pathway inhibitors against the *ALDH1A1*, *SOX2*, and *POU5F1* genes.

**Figure 8:**
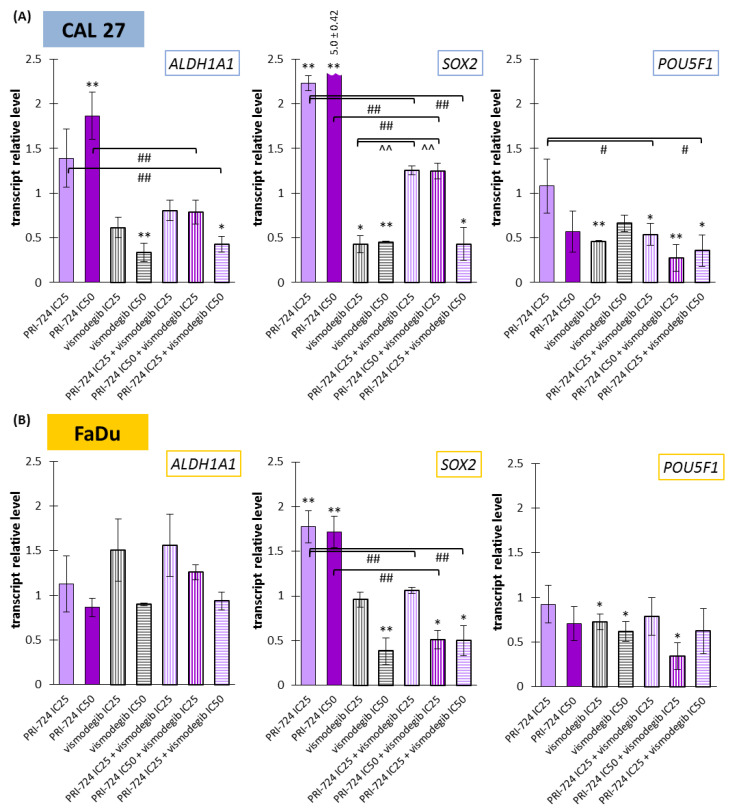
The effect of PRI-724 and vismodegib on the relative transcript levels of *ALDH1A1*, *SOX2*, and *POU5F1* genes in CAL 27 (**A**) and FaDu (**B**) cells. Mean values ± SD from three independent experiments with three replicates per R-T PCR reaction are shown. The level of DMSO-treated cells was considered to be 1. The asterisk (*) above the bar denotes statistically significant changes in comparison to control (* *p* < 0.05, ** *p* < 0.01), (#) denotes statistically significant changes in comparison to PRI-724 (# *p* < 0.05, ## *p* < 0.01), and (^) denotes statistically significant changes in comparison to vismodegib (^^ *p* < 0.01).

On the one side, PRI-724 upregulated the expression of *ALDH1A1* and *SOX2* in CAL 27 cells (Figure 8A) and *SOX2* in FaDu cells (Figure 8B). In other cases, there was no significant change. On the other side, vismodegib in one or both concentrations was able to decrease the expression of the evaluated genes, with the exception of *ALDH1A1* in FaDu cells.

The expression of *ALDH1A1* was unchanged in FaDu cells, but in CAL27 cells, a reduction was present for the mixture of PRI-724 IC25 and vismodegib IC50. Two other combinations represented balanced effects on gene expression, i.e., between an induced level for PRI-724 and a decreased level for vismodegib. The co-treatment of CAL27 cells with PRI-724 IC25 and vismodegib IC50 decreased the expression of *SOX2* compared to the control. Due to intensively upregulated expression of *SOX2* under the influence of PRI-724 and significantly downregulated expression after vismodegib treatment, mixtures of PRI-724 in both concentrations with vismodegib IC25 resulted in an intermediate level of expression. However, the effect of vismodegib IC50 was dominant in mixtures. For FaDu cells, the effects of combinations were similar to those seen for vismodegib alone, but the mixture of PRI-724 IC50 with vismodegib IC25 presented the opposite action compared to PRI-724 used individually.

Finally, *POU5F1* expression was downregulated by approximately 70% after exposure of CAL 27 and FaDu cells to PRI-724 IC50 and vismodegib IC25.

## 3. Discussion

Molecularly targeted therapy based on the inhibition of EGFR signaling did not meet expectations for the improvement of the clinical fate of patients with HNSCC [26]. Thus, new potential targets have been intensively searched for. Wnt canonical signaling is considered a potential target, due to various genetic and epigenetic dysregulations affecting its activity, which are found in HNSCC cells, also at pre-clinical stages [8,10,27]. The inhibition of the nuclear interaction between CBP acetyltransferase and β-catenin translocated from the cytoplasm to the nucleus was described in our previous research as the best way to target this pathway [15]. Particularly, a small-molecule PRI-724 inhibitor was tested. Others have also reported the beneficial effects of this chemical. PRI-724 showed positive results in early-stage clinical trials with advanced ovarian cancer or pancreatic adenocarcinoma patients. Moreover, it showed therapeutic potential in soft tissue sarcomas, leading to a reduction in cancer cell viability, disruption of cell cycle progression, and promotion of apoptosis in pre-clinical tests [28]. Results from the same study also pointed to the synergistic activity of the combination of PRI-724 with standard chemotherapeutics such as doxorubicin or trabectedin. In other research, PRI-724 blocked the proliferation and formation of fibrolamellar hepatocellular carcinoma organoids [29]. In addition, the antitumor properties of PRI-724 were found in the cisplatin-resistant human embryonal carcinoma NTERA-2 cells [30] or in the human neuroendocrine tumor BON1, QGP-1, and NCI-H727 cells [31]. In acute myeloid leukemia, the *FLT3* gene (which encodes a class III receptor tyrosine kinase that regulates hematopoiesis) is frequently mutated, and *FLT3*-mutant cells are able to promote the Wnt/β-catenin pathway by activating Integrin αvβ3/PI3K/Akt/GSK-3β signaling [32]. The inhibition of FLT3 signaling by sorafenib or quizartinib with the concomitant use of PRI-724 to block the transcriptional activity of β-catenin improved the effects against acute myeloid leukemia stem cells [33]. Indeed, monotherapy based on PRI-724 and other Wnt pathway inhibitors is insufficient in molecularly diverse tumor cells, and thus PRI-724 should rather be combined with chemicals affecting other molecular targets.

Targeting the Wnt/β-catenin pathway exerted better effects in HPV-positive than in HPV-negative HNSCC tumors, in which a combinatorial treatment could have more prominent effects [34]. Interestingly, stronger anti-neoplastic and radiosensitizing effects of β-catenin inhibition were shown in HPV-negative cells, but greater anti-migratory potential was detected in HPV-positive HNSCC cells [35]. The different profiles of action against HPV-positive and HPV-negative cancer cells might be related to the diverse molecular features of these etiologically distinct groups of tumors [36]. Moreover, we also detected therapeutic crosstalk between PI3K/Akt/GSK-3β signaling and the Wnt pathway in HNSCC. In this regard, the mixture of an Akt kinase inhibitor together with either a Porcupine inhibitor (IWP-O1) or an inhibitor of CBP/β-catenin interaction (PRI-724) potentiated cell viability reduction in tongue SCC cells growing in 3D-culture as spheroids [37].

Wnt signaling is one of the master regulators of stem cells during embryogenesis and in selected adult tissues, but also during carcinogenesis [38]. Indeed, targeting cancer stem cells by inhibiting the Wnt canonical pathway is one of the possibilities to cure colorectal cancer [39], but obviously it should also be tested in all Wnt signaling-dependent tumors. Hedgehog (Hh) signaling is another pathway controlling SC and CSC [38,39]. Since 2012, vismodegib, an inhibitor of the Hh pathway, has been registered for the treatment of basal cell carcinoma (BCC) [23]. Although BCC and HNSCC are different types of tumors, some molecular similarities can be observed. In a case of sporadic BCC in an 80-year-old man, both Wnt and Hh signaling pathways were overexpressed [40]. In addition, the insulin-like growth factor 2 mRNA-binding protein 1 (IGF2BP1), a target gene of Wnt signaling, was overexpressed and further promoted the transcription level of Gli1, a transcription factor in the Hh pathway. More than twenty years ago, Mullor et al. (2001) described the ability of Gli1 to activate the expression of several Wnt ligands in early frog embryos [41]. Moreover, in RTOG10 cells, Gli1 induced the transformation of epithelial cells via the induction of Snail, a repressor of E-cadherin, which released β-catenin from the cell membrane compartment and induced its translocation to the cell nucleus [42].

Interactions between Wnt and Hh pathways and their special role in cancer stem cells were the basis of the idea to evaluate the effects of the simultaneous treatment of HNSCC cells by PRI-724 and vismodegib. Targeting EGFR and related signaling pathways led to limited effects against HNSCC [3]. For this reason, we previously performed experiments on CAL 27 and FaDu cells to test the effects of erlotinib (an EGFR inhibitor) and HS-173 (a PI3K kinase inhibitor) separately and in combination with inhibitors of KDM4 and KDM6 histone demethylases [6]. Individual compounds had limited influence, e.g., on the induction of apoptosis. However, cell death was synergistically potentiated after the addition of KDM inhibitors. In the current study, we used erlotinib and HS-173 in mixtures with PRI-724 to compare their effects with the anti-neoplastic influence of the combined use of PRI-724 and vismodegib. Importantly, recent studies have shown that PRI-724, vismodegib, and erlotinib were less active in non-cancerous HaCaT keratinocytes, suggesting some selectivity of these chemicals towards cancer cells [43,44,45].

The sensitivity of tongue cancer-derived CAL 27 cells and hypopharyngeal cancer-derived FaDu cells to Wnt and Hh pathway inhibitors was similar at sub-toxic concentrations (IC25). In turn, lower concentrations of both chemicals were needed to reach a 50% reduction in viability in CAL 27 cells compared to FaDu cells. Interestingly, the combination of PRI-724 and vismodegib had a rather weak synergistic effect on CAL 27 cells viability, while this combination of compounds at higher concentrations showed synergism in FaDu cells. Thus, hypopharyngeal tumor cells were more susceptible to the dual inhibition of Wnt and Hh signaling. A similar correlation was seen for the mixture of PRI-724 and erlotinib, whereas PRI-724 and the PI3K kinase inhibitor HS-173 acted highly synergistically in the full range of concentrations.

The incubation of CAL 27 and FaDu cells with vismodegib for 48 h resulted in the enrichment of the G1/G0 population of cells with a concomitant slight induction of the percentage of apoptotic cells. These results are generally consistent with the data generated by Freitas et al. (2020). They compared the effects of Hh pathway inhibitors vismodegib and itraconazole and conventional chemotherapeutics doxorubicin (DOX) and 5-fluorouracil (5-FU) used individually. Uncombined vismodegib clearly reduced the viability of CAL 27 cells after 48 h of incubation, but pro-apoptotic and anti-proliferative effects were much weaker than in the case of DOX and 5-FU [46].

The effectiveness of vismodegib was previously assessed in the basal cell carcinoma line BCC-1 and in the tongue cancer line SCC-25 in combination with irradiation. In both cellular models, the treatment resulted in reduced cell proliferation and radiosensitization [47]. In another study, vismodegib mixed with docetaxel, cisplatin, or cetuximab additively enhanced the anti-neoplastic effects in HNSCC samples derived ex vivo [48]. In our study, mixtures of the Wnt/β-catenin pathway inhibitor with vismodegib caused the accumulation of hypopharyngeal cancer cells in G1/G0 phases. In CAL 27 cells, the combination of PRI-724 IC50 and vismodegib IC25 enriched rather S phase cells, similarly to the sole use of PRI-724 IC50. A more significant induction of apoptosis appeared upon individual exposure of cells to PRI-724 and vismodegib, and this effect was lost in the mixture with vismodegib at higher concentrations. We can assume that the combination of Wnt and Hh signaling inhibition has better anti-proliferative than pro-apoptotic effects. A higher induction of apoptosis was detected in the other tested groups. PRI-724 combined with erlotinib or HS-173 presented a significant increase in apoptotic cell populations, although its individual use had rather low efficacy [6]. In cell cycle analysis, erlotinib had a dominant influence in combination with PRI-724. However, despite no effect of individually applied HS-173, its combination with PRI-724 was able to block cell cycle in S and G2/M phases in FaDu cells. Because of the synergistic effect on the reduction of viability and the highest induction of apoptotic death of HNSCC cells (among the evaluated combinations), the simultaneous disruption of Wnt and PI3K pathways seems to be an interesting option for further research in the field of pharyngeal SCC tumors.

The inhibition of signaling pathways may also have an influence on cell migration. Thus, we performed the scratch assay for all the tested inhibitors used individually and in combinations. CAL 27 and FaDu cells were susceptible to the reduction of migration rate, and vismodegib demonstrated better activity than the other chemicals. In contrast, the inhibition was not so significant in lung SCC H1703 and H12170 cells [49]. Furthermore, co-treatment of HNSCC cells with Wnt and Hh, EGFR, or PI3K inhibitors significantly reduced their migration. PRI-724 with vismodegib at a lower concentration (IC25) exerted a highly synergistic reduction of migration rate, especially in FaDu cells. Therefore, although the joint inhibition of Wnt/β-catenin and Hh signaling is unable to strongly induce cell death, it can intensively affect HNSCC proliferation and migration. In addition, PRI-724 with erlotinib also acted in a synergistic way. Thus, the potential of EGFR inhibitors in HNSCC tumors can still be improved.

In HNSCC tumors, cancer stem cells are present and can be detected by increased expression of, e.g., ALDH1 (*ALDH1A1* gene), SOX2 (*SOX2* gene), and OCT4 (*POU5F1* gene) [50,51,52]. We checked the expression levels of these genes among HNSCC patients based on data available from The Cancer Genome Atlas. We observed a decreased mRNA level for ALDH1 and SOX2, but an increased transcript level for OCT4. Moreover, advanced grade 4 tumors (potentially richer in the CSC population) showed the highest expression of those genes. SOX2 and OCT4 mRNA expression was increased compared to normal tissue. We assumed that in cancer cells, which contain a sub-population of CSC, the downregulation of CSC markers should be more visible in the case of over-expressed transcripts, like OCT4 mRNA. We wanted to preliminary check if Wnt/β-catenin and/or Hedgehog signaling inhibitors are able to modulate the transcript level of those CSC-related genes in CAL 27 and FaDu cells. Indeed, in FaDu cells, *ALDH1A1* lacked any significant changes. Surprisingly, incubation with the Wnt signaling inhibitor induced the expression of those CSC markers, but in contrast, the inhibition of Hh decreased their transcript level. The opposite effect for PRI-724 probably results from differences in time to evoke and sustain molecular changes after Wnt or Hh pathway inhibition. Hypothetically, gene expression upregulation after exposure to PRI-724 might be a secondary effect after the initial decrease in expression because, in other research, CBP/β-catenin targeting had inhibitory effects in lung cancer stem cells [53]. However, the gene encoding the OCT4 protein, which was overexpressed in patient-derived samples, was importantly downregulated in response to single and combined inhibitors. The expression of OCT4 is directly regulated by β-catenin in HNSCC stem-like cells, and higher levels of both OCT4 and β-catenin correlate with the worst prognosis for patients [54], so our results from CAL 27 and FaDu cells point to beneficial effects of the applied combination of inhibitors. Vismodegib was shown to reduce OCT4 (*POU5F1*) expression in biliary tract cancer Mz-ChA-1 and Sk-ChA-1 cells [55]. What is noteworthy is that in our study, vismodegib potently counteracted the inducing effect of PRI-724 in relation to *ALDH1A1* and *SOX2* expression, and the combinations showed more beneficial effects. These results are in line with the synergistic effects of Wnt and Hh pathway co-inhibition with respect to cell migration, cell cycle disruption, and viability. However, in future studies, the direct impact of PRI-724 and vismodegib on the CSC subpopulation must be evaluated.

The balancing of the exerted effects and the potentialization of activity speak in favor of using the combinations of PRI-724 with not only vismodegib but also with erlotinib and HS-173, and they invite more detailed subsequent studies. It should also be remembered that the molecular landscape of cancer cells differs between patients and is dynamic. In the future, by working with, e.g., ex vivo cultured cancer cells derived from HNSCC patients, the different profiles/endotypes of tumors might be identified. Artificial neural network analysis [56] based on new data can help establish the association between tumor profiles and benefit from the combinations proposed in this article.

## 4. Materials and Methods

### 4.1. Cells and Culture Conditions

Commercially available HNSCC cells were used in the experiments: CAL 27 cells derived from tongue cancer and FaDu cells derived from hypopharyngeal cancer were both purchased from the American Type Culture Collection (ATCC).

The cells were grown in high-glucose DMEM medium (Biowest, Nuaillé, France), supplemented with 10% FBS (EURx, Gdańsk, Poland) and 1% antibiotic solution (penicillin and streptomycin; Biowest, Nuaillé, France), and were cultured under standard conditions (37 °C, 5% CO_2_, 95% humidity) in an incubator (Memmert, Schwabach, Germany).

### 4.2. Chemicals and Cell Viability Assay

Four small-molecule inhibitors of signaling pathways were used in the research. The Wnt canonical signaling inhibitor PRI-724, the Hedgehog signaling inhibitor vismodegib, and the EGF receptor intracellular domain inhibitor erlotinib were ordered from Selleck Chemicals (Pittsburgh, PA, USA), while the inhibitor of the p110α domain of PI3K was purchased from Sigma-Aldrich (St. Louis, MO, USA). Stock solutions of the compounds were prepared in DMSO and stored in aliquots at −20 °C.

Cells (10^4^/well) were seeded into black 96-well plates. After 24 h of pre-incubation, a fresh medium containing different concentrations of the compounds (single or in combination) was added. Control cells were treated with vehicle (DMSO < 0.2%). After 48 h, cells were washed with PBS buffer, and fresh medium containing 1 μg/mL resazurin (Sigma-Aldrich, St. Louis, MI, USA) was added. After 2 h of additional incubation, the fluorescence was measured (ex—530 nm, em—590 nm) using an Infinite M200 multiplate reader (Tecan, Grödig, Austria). Three independent experiments were performed, with four separate replicates per experiment.

The influence of the combination of PRI-724 inhibitor with other compounds in relation to cell viability was determined by the evaluation of the Combination Index (CI) using the CompuSyn software (downloaded from the website www.combosyn.com on 31 May 2021) [57]. The synergistic action of the chemicals in combinations was identified when CI ˂ 1.

### 4.3. Analysis of the Cell Cycle Distribution

The effect of single compounds and combinations of PRI-724 with other inhibitors on cell cycle distribution was analyzed using the Muse^®^ Cell Cycle Kit (Merck, Darmstadt, Germany) according to the manufacturer’s protocol. Briefly, 2 × 10^5^ cells per well were seeded in 6-well plates. After 24 h of pre-incubation, the growth medium was replaced with fresh medium (2 mL per well) containing IC25 and/or IC50 concentrations of the compounds, and the cells were incubated for an additional 48 h. The cells incubated with DMSO served as a negative control, while cells incubated with 100 nM topotecan (Sigma-Aldrich, St. Louis, MI, USA) served as a positive control for cell cycle arrest. After incubation, cells were collected by trypsinization, washed with PBS buffer, fixed in ice-cold 70% ethanol, and stored overnight at −20 °C. The next day, fixed cells were collected by centrifugation and washed with PBS buffer. The distribution of cells depending on cell cycle phase (G1/G0, S, G2/M) was analyzed with the Muse^®^ Cell Analyzer (Merck, Darmstadt, Germany) after staining with propidium iodide solution in the presence of RNase A for 30 min. The data were analyzed using Muse^®^ 1.5 Analysis software (Merck, Darmstadt, Germany). All the experiments were done in triplicate.

### 4.4. Analysis of Apoptosis

The externalization of phosphatidylserine was applied as a marker of apoptotic cells and analyzed using the Muse^®^ Annexin V and Dead Cell Kit (Merck, Darmstadt, Germany) according to the manufacturer’s protocol. The 7-aminoactinomycin D (7-AAD) stain was applied as a counterstain to Annexin V to discriminate between early and late apoptotic cells. Briefly, 2 × 10^5^ cells per well were seeded in 6-well plates. After 24 h of pre-incubation, the growth medium was replaced with fresh medium (2 mL per well) containing IC25 and/or IC50 concentrations of the compounds, and the cells were incubated for an additional 48 h. The cells incubated with DMSO served as a negative control, while cells incubated with 100 nM topotecan (Sigma-Aldrich, St. Louis, MI, USA) served as a positive control for active apoptosis. Subsequently, the cells were collected and stained with Annexin V and 7-AAD in a culture medium solution. After 20 min of incubation, the cells were analyzed by flow cytometry on the Muse^®^ Cell Analyzer (Merck, Darmstadt, Germany), and the data were further evaluated using Muse^®^ 1.5 Analysis software (Merck, Darmstadt, Germany).

### 4.5. Analysis of Cell Migration Using the Scratch Wound Assay

Cell migration was assessed by the scratch assay. Cells (5 × 10^5^) were seeded into 24-well plates and grown overnight to reach confluency. A scratch was performed using a 10 μL tip. Wells were rinsed twice with PBS buffer to remove detached cells, and fresh medium with a reduced FBS concentration (0.5%) containing IC25 and/or IC50 concentrations of the compounds was added. Subsequently, representative microscopic photographs of scratch areas were taken by a JuLI FL microscope (NanoEntek, Seoul, Republic of Korea) directly after the addition of compounds (0 h) and after 18 h. Area covered by cells (%) was assessed using JuLI FL software, and the difference in the coverage of the growth area by cells between 18 h and 0 h time points was calculated for each well. The experiment was repeated four times. The relative effect of the tested compounds on cell migration (*RM*) was calculated using the following formula:

(1) $$RM=\frac{tested\;compound\;area\;after\;18\;h-tested\;compound\;area\;after\;0\;h}{negative\;control\;area\;after\;18\;h-negative\;control\;area\;after\;0\;h}\times 100\%$$

The combinatorial effects of the compounds on cell migration were evaluated using the modified Bürgi formula [58,59] in order to assess the type of interaction between chemicals used in IC25 and/or IC50 concentrations. The *q* factor was calculated using the equation:

(2) $$q=\frac{P_{mix}}{\left(P_{1}+P_{2}\right)-(P_{1}\times P_{2})}$$

where *P*_1_ denotes the effect of PRI-724 treatment, *P*_2_ denotes the effect of a second compound (vismodegib, erlotinib, or HS-173) treatment, and *P_mix_* denotes the effect of a combination of chemicals. Antagonistic effects were detected when *q* < 0.85, while synergistic effects (potentiation of action) were detected when *q* > 1.15. The compounds were considered to act independently of each other (additive effects) if *q* was in the range 0.85–1.15.

### 4.6. Expression Analysis Based on TCGA

In Figure 1 and Figure 7, we present the analysis of gene expression data available from The Cancer Genome Atlas (TCGA). We used the UALCAN tool (https://ualcan.path.uab.edu, accessed on 11 April 2023 and 7 June 2023) [24,25] to show differences in the expression of genes related to the Wnt signaling pathway between healthy controls and HNSCC patients depending on the histological cancer grade (Figure 1) or genes related to cancer stem cells (Figure 7) between healthy controls and HNSCC patients and among HNSCC patients depending on the histological cancer grade (1–4).

### 4.7. Isolation of RNA, Reverse Transcription, and Quantitative Real-Time PCR

Total RNA was isolated from cells treated with the compounds for 48 h using the Universal RNA Purification Kit (EURx, Gdańsk, Poland), and samples were subjected to reverse transcription by the NG dART RT Kit (EURx, Gdańsk, Poland), according to the manufacturer’s instructions.

For the quantitative R-T PCR analyses, the SG qPCR Master Mix (EURx, Gdańsk, Poland) and LightCycler 96 (Roche, Basel, Switzerland) were used. The initial enzyme activation at 95 °C lasted 10 min and was followed by 40 three-step cycles consisting of denaturation at 95 °C for 15 s, primer annealing at 56 °C for 30 s, elongation at 72 °C for 30 s with fluorescence measurement, and subsequently followed by melting curve analysis. The relative mRNA expression of Aldehyde dehydrogenase 1—ALDH1 (*ALDH1A1* gene), SRY (sex determining region Y)-box 2—SOX2 (*SOX2* gene), and Octamer-binding transcription factor 4—OCT4 (*POU5F1* gene) was determined. The expression of the TATA-box-binding protein (*TBP* gene) was used to normalize the data. The ΔΔCt method was used for fold-change quantification. Three independent experiments were performed with three technical repeats for each sample during R-T PCR. The sequences of primers used in the research are listed in Table 2.

### 4.8. Statistical Analysis

To analyze the significance of differences between controls and signaling pathway inhibitors: PRI-724, vismodegib, erlotinib, and HS-173, a one-way ANOVA test with the Tukey post hoc test was performed, with *p* < 0.05 considered significant. The analyses were performed using STATISTICA software (version 11.0).

## 5. Conclusions

This in vitro study implicated new directions in the development of molecularly targeted treatment of head and neck squamous carcinoma. The active status of the Wnt/β-catenin pathway stimulates the progression of HNSCC cells, but as previously reported, anti-cancer effects should be improved by co-targeting other signaling pathways important for HNSCC carcinogenesis. According to our results, Wnt/β-catenin and Hh signaling can interact with each other, so their concomitant inhibition presented in this research might be a good strategy to attenuate the growth of HNSCC tumors. Simultaneous inhibition of the Wnt and Hh pathways caused pronounced effects concerning decreased proliferation and migration. CAL 27 and FaDu cells, although coming from different locations of HNSCC, were in general both sensitive to the combinatorial inhibition of Wnt/β-catenin and Hh pathways. Notably, the simultaneous blockade of the EGFR-related pathways and Wnt/β-catenin pathway also presented promising results. The migration of HNSCC cells was diminished by mixtures of PRI-724 and erlotinib, or HS-173, to a lesser extent. However, the anti-proliferative activity and more robust induction of apoptosis can be the basis for the improvement of the anti-neoplastic effects of EGFR and PI3K inhibitors.

**Figure 9:**
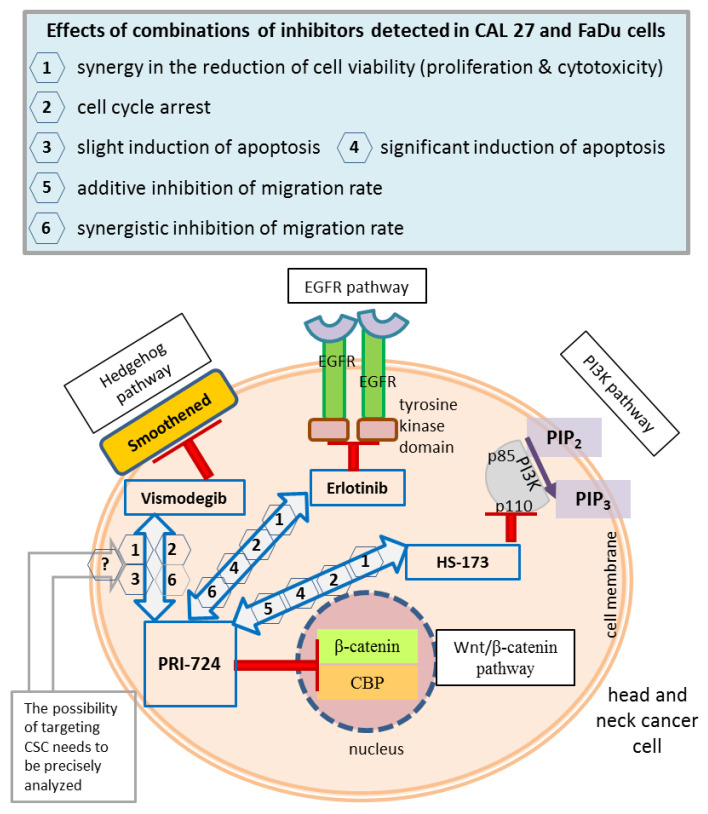
A summary of the main concepts and results of the study. Wnt/β-catenin signaling was inhibited by the PRI-724 small molecule, which targets the interaction between nuclear β-catenin and the CREB binding protein (CBP). PRI-724 was combined with the Hedgehog pathway inhibitor vismodegib, which blocks the Smoothened protein; the epidermal growth factor receptor (EGFR) pathway inhibitor erlotinib, which interacts with the cytoplasmic tyrosine kinase domains of EGFR; and the phosphoinositide 3-kinase (PI3K) inhibitor HS-173, which targets the p110α domain of PI3K. The effects detected in CAL 27 and FaDu cells after treatment with each combination of inhibitors used in this research are listed in the frame (upper panel), and appropriate numbers appear next to arrows denoting compound mixes in the head and neck cancer cell schematic representation. CSC, cancer stem cells; PIP2, phosphatidylinositol 4,5-bisphosphate; PIP3, phosphatidylinositol 3,4,5-trisphosphate.

The main concepts of signaling pathway co-inhibition and the findings of this research are summarized in Figure 9.

The results are preliminary, and thus the full potential of the combinations of the studied chemicals should be further evaluated. The prospective research will cover testing the discussed molecularly targeted drug combinations in other cell lines to evaluate the generalizability of current results and the mechanism of action. More advanced in vitro models will be introduced, including three-dimensional cultures of cancer cells (spheroids, organoids) and co-cultures with fibroblasts to observe micro-environmental interactions between signaling pathways. Ex vivo culture of patient-derived cancer cells might be the final model for evaluating this treatment concept before in vivo experiments.

Wnt/β-catenin and Hh pathways are both related to the control of CSCs. The expression of exemplary CSC-related genes among HNSCC patients increases in parallel with tumor stage. Concomitant inhibition of Wnt/β-catenin and Hh pathways was able to partly reduce their transcript levels in CAL 27 and FaDu cells. Therefore, further experiments should also verify the usefulness of co-targeting those two signaling pathways for affecting CSCs in HNSCCs. Such an effect can be crucial for preventing cancer recurrence, which is thought to be tightly connected with the subpopulation of cancer stem cells resistant to current therapy.

## Figures and Tables

**Table 1:** The IC25 and IC50 values for CAL 27 and FaDu cells based on the resazurin assay.

Compound	CAL 27		FaDu	
	IC25 [µM]	IC50 [µM]	IC25 [µM]	IC50 [µM]
PRI-724	2.6	8.3	2.1	14.6
vismodegib	18.0	39.4	21.0	74.8
erlotinib	0.3 *	2.9 *	0.6 *	7.0 *
HS-173	0.09 *	0.2 *	0.125 *	0.8 *

* IC25 and IC50 values determined by the resazurin assay and previously reported [6].

**Table 2:** Primers used in the R-T PCR.

Gene	Forward Primer	Reverse Primer
*ALDH1A1*	5′CTGTCCTACTCACCGATT	5′CCTCCTTATCTCCTTCTTCTA
*POU5F1*	5′GGTTCTATTTGGGAAGGTAT	5′CATGTTCTTGAAGCTAAGC
*SOX2*	5′ATGGTTGTCTATTAACTTGT	5′TCTCTCCTCTTCTTTCTC
*TBP*	5′GGCACCACTCCACTGTATC	5′GGGATTATATTCGGCGTTTCG

## Data Availability

Data are contained within the article and Supplementary Materials.

## Supplementary Materials

The following supporting information can be downloaded at: https://www.mdpi.com/article/10.3390/ijms241310448/s1.
