# Supplementary material for: Combinations of PRI-724 Wnt/β-Catenin Pathway Inhibitor with Vismodegib, Erlotinib, or HS-173 Synergistically Inhibit Head and Neck Squamous Cancer Cells
Source: Int J Mol Sci. 2023 Jun 21;24(13):10448. doi: 10.3390/ijms241310448 (PMC10341739; doi:10.3390/ijms241310448)
Supplement: Supplementary file 1 [file ijms-24-10448-s001.zip › Supplementary Materials.pdf]

Supplementary materials

# Combinations of PRI-724 Wnt/β-Catenin Pathway Inhibitor with Vismodegib, Erlotinib, or HS-173 Synergistically Inhibit Head and Neck Squamous Cancer Cells

**Robert Kleszcz [1,*], Mikołaj Frąckowiak [1], Dawid Dorna [1] and Jarosław Paluszczak [1]**

[1] Department of Pharmaceutical Biochemistry, Poznan University of Medical Sciences, 4, Święcickiego Str., 60-781 Poznań, Poland; kleszcz@ump.edu.pl (R.K.), mikfrackowiak97@gmail.com (M.F.), dawid.dorna97@gmail.com (D.D.), paluszcz@ump.edu.pl (J.P)

* Correspondence: kleszcz@ump.edu.pl

**Table S1. Detailed concentrations and effects of viability inhibition used for analysis in the CompuSyn software for individual compounds and indicated mixtures.**

| concentrations [µM] | #1 | #2 | #3 | #4 | #5 | #6 |
|---|---|---|---|---|---|---|
| PRI-724 | 1.00 | 2.00 | 5.00 | 10.00 | 20.00 | 40.00 |
| erlotinib | 1.00 | 2.00 | 5.00 | 10.00 | 20.00 | 40.00 |
| HS-173 | 0.05 | 0.10 | 0.25 | 0.50 | 1.00 | 2.00 |
| vismodegib | 1.00 | 2.00 | 5.00 | 10.00 | 20.00 | 40.00 |
| **effects* in CAL 27 cells** | **#1** | **#2** | **#3** | **#4** | **#5** | **#6** |
| PRI-724 (R) | 0.170 | 0.186 | 0.433 | 0.535 | 0.572 | 0.780 |
| erlotinib (E) | 0.354 | 0.436 | 0.533 | 0.605 | 0.702 | 0.739 |
| HS-173 (H) | 0.150 | 0.295 | 0.625 | 0.855 | 0.890 | 0.900 |
| vismodegib (V) | 0.020 | 0.047 | 0.102 | 0.161 | 0.271 | 0.507 |
| R + E (1:1) | 0.416 | 0.630 | 0.686 | 0.747 | 0.820 | 0.954 |
| R + H (1:0.05) | 0.252 | 0.506 | 0.747 | 0.792 | 0.813 | 0.937 |
| R + V (1:1) | 0.195 | 0.214 | 0.470 | 0.537 | 0.709 | 0.775 |
| **effects* in FaDu cells** | **#1** | **#2** | **#3** | **#4** | **#5** | **#6** |
| PRI-724 (R) | 0.196 | 0.245 | 0.410 | 0.481 | 0.543 | 0.774 |
| erlotinib (E) | 0.320 | 0.412 | 0.470 | 0.606 | 0.629 | 0.696 |
| HS-173 (H) | 0.120 | 0.204 | 0.388 | 0.452 | 0.531 | 0.736 |
| vismodegib (V) | 0.020 | 0.042 | 0.074 | 0.118 | 0.247 | 0.316 |
| R + E (1:1) | 0.222 | 0.508 | 0.589 | 0.685 | 0.711 | 0.862 |
| R + H (1:0.05) | 0.324 | 0.556 | 0.662 | 0.739 | 0.821 | 0.904 |
| R + V (1:1) | 0.051 | 0.123 | 0.359 | 0.514 | 0.689 | 0.924 |

* effect of viability reduction is calculated as 1 – viability (in scale 1 = 100%, 0 = 0%)
